# Chitosan and Its Derivatives for Application in Mucoadhesive Drug Delivery Systems

**DOI:** 10.3390/polym10030267

**Published:** 2018-03-05

**Authors:** Twana Mohammed M. Ways, Wing Man Lau, Vitaliy V. Khutoryanskiy

**Affiliations:** 1 Reading School of Pharmacy, University of Reading, Whiteknights, Reading RG6 6AD, UK; t.m.m.ways@pgr.reading.ac.uk; 2 School of Pharmacy, Faculty of Medical Sciences, Newcastle University, Newcastle upon Tyne NE1 7RU, UK; wing.lau@newcastle.ac.uk

**Keywords:** chitosan derivatives, mucosal drug delivery, mucoadhesion, trimethyl chitosan, thiolated chitosan, chitosan-catechol, acrylated chitosan

## Abstract

Mucoadhesive drug delivery systems are desirable as they can increase the residence time of drugs at the site of absorption/action, provide sustained drug release and minimize the degradation of drugs in various body sites. Chitosan is a cationic polysaccharide that exhibits mucoadhesive properties and it has been widely used in the design of mucoadhesive dosage forms. However, its limited mucoadhesive strength and limited water-solubility at neutral and basic pHs are considered as two major drawbacks of its use. Chemical modification of chitosan has been exploited to tackle these two issues. In this review, we highlight the up-to-date studies involving the synthetic approaches and description of mucoadhesive properties of chitosan and chitosan derivatives. These derivatives include trimethyl chitosan, carboxymethyl chitosan, thiolated chitosan, chitosan-enzyme inhibitors, chitosan-ethylenediaminetetraacetic acid (chitosan-EDTA), half-acetylated chitosan, acrylated chitosan, glycol chitosan, chitosan-catechol, methyl pyrrolidinone-chitosan, cyclodextrin-chitosan and oleoyl-quaternised chitosan. We have particularly focused on the effect of chemical derivatization on the mucoadhesive properties of chitosan. Additionally, other important properties including water-solubility, stability, controlled release, permeation enhancing effect, and in vivo performance are also described.

## 1. Introduction

Mucus is a viscoelastic gel lining the mucosal tissues exposed to the external environment including gastrointestinal, respiratory, and reproductive tracts and the eyes [1,2]. It is mainly composed of water (~90 to 98%), mucins (0.2–5% *w*/*w*), salts (~0.5 to 1.0% *w*/*w*), proteins (~0.5% *w*/*v*), cells and cellular debris, DNA, bacteria and lipids [1,2,3,4,5,6,7]. Mucins are the main component of the mucus, which are glycoproteins responsible for its gel-like characteristics. These glycoproteins are made of protein core to which carbohydrate side chains are covalently attached via *O*-glycosidic linkages [8,9].

Conventional (non-mucoadhesive) formulations lack the ability to withstand the strong involuntary muscular movement as well as the extensive washing effect by certain body fluids available, e.g., in the gastrointestinal lumen, ocular surface, urinary bladder and other mucosal surfaces. This limitation leads to the loss of a substantial amount of the administered drugs at the site of application/absorption. This may not only result in the overall increased cost of the treatment courses; it can also lead to the failure of therapy as effective drug concentration cannot be reached. This is especially more important in case of drugs such as antibiotics as amount lower than minimum inhibitory concentration probably leads to intractable complications including bacterial resistance. Mucoadhesive drug delivery systems are advantageous as they can adhere to the mucus layer of the mucous membrane. The adhesion of the delivery systems to mucosa (defined as mucoadhesion) increases the residence time of drugs, increases the concentration gradient, and protects the vulnerable small molecular weight drugs as well as peptide-based drugs. The overall effects could lead to controlled drug release, prolongation of therapeutic effects, enhancement in the bioavailability, cost-effective treatment, and improved patient compliance [2,9,10,11,12]. However, transmucosal drug delivery systems often have poor residence on mucosal surfaces, which justifies the need for novel mucoadhesive materials. 

**Figure 1:**
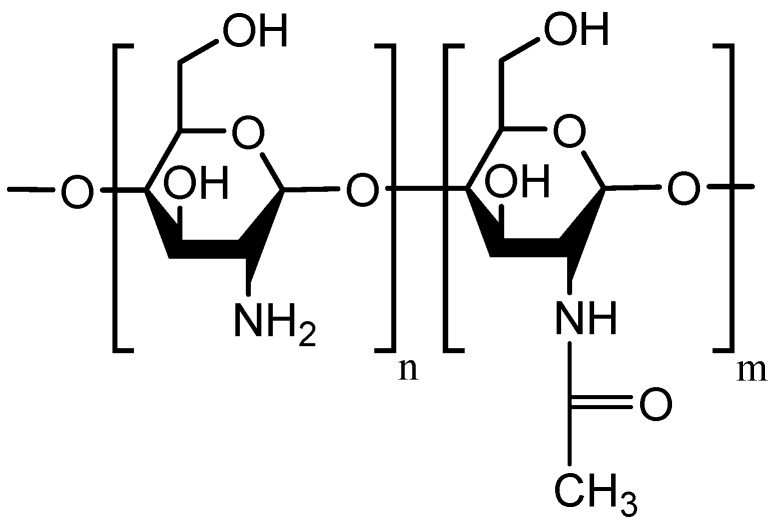
Chemical structure of chitosan.

Various polymers have been used in the formulation of mucoadhesive delivery systems. Among them, chitosan and its derivatives are listed at the top [2,4,13,14,15,16]. Chitosan is a polysaccharide composed of *N*-acetyl-d-glucosamine and d-glucosamine and its units linked by 1-4-β-glycosidic bonds (Figure 1). It can be prepared by deacetylation of chitin in basic media [17,18]. Chitin is the second most abundant polysaccharide in nature, while cellulose is the most abundant [18]. Crustaceans produce chitin in their shells and plants produce cellulose in their cell walls. Therefore, these two polysaccharides impart structural integrity and protection to animals and plants [19].

Chitosan has –OH and –NH_2_ groups leading to the capability of forming hydrogen and covalent bonding. This characteristic results in the possibilities of various chitosan chemical derivatization. These functional groups also play an essential role in the solubility character of chitosan macromolecules. At low pH, the amino groups undergo protonation, which makes chitosan macromolecules positively charged. This cationic nature provides strong electrostatic interaction with negatively charged components of mucus including sialic acid as well as epithelial surfaces [2,8,15,20,21,22,23]. Hydrogen bonding and hydrophobic interaction also play important role in the mucoadhesion of chitosan [15].

**Figure 2:**
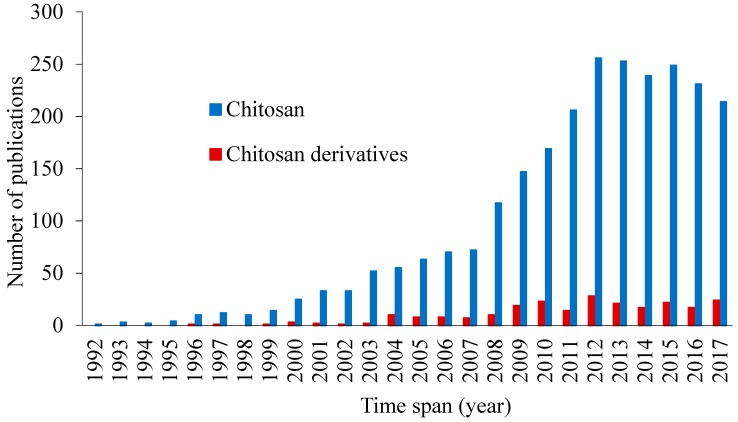
Number of publications related to mucoadhesive properties of chitosan and chitosan derivatives, source: SciFinder, keywords: chitosan or chitosan derivatives and mucoadhesion, retrieved on 24 November 2017.

The derivatization of chitosan to improve its mucoadhesive properties has been considered in several publications (Figure 2). Some chitosan and its derivatives have shown potential in preclinical and clinical investigations for applications in transmucosal drug delivery (e.g., ChiSys^®^ as a platform for nasal vaccination [24] and Lacrimera^®^ eye drops [25]). However, there is still lack of review articles analyzing recent studies on the mucoadhesive applications of chitosan derivatives. In this review, we report various chitosan derivatives with potential applications as mucoadhesive materials. This review, however, does not consider any physical mixtures of chitosan or salt forms, which are discussed in several previous publications [26,27].

## 2. Chitosan as a Mucoadhesive Material

Chitosan has been widely used in various biomedical and drug delivery areas because of its low toxicity, biocompatibility, antimicrobial activity, mucoadhesive properties and permeation enhancing effects [4,15,18,28,29,30,31,32]. It has been extensively studied as a potential excipient for the oral delivery of peptides [33]. Alonso and co-workers found that chitosan nanocapsules enhanced and prolonged intestinal absorption of salmon calcitonin because of their mucoadhesive properties and strong interactions with the intestinal barrier [34].

**Figure 3:**
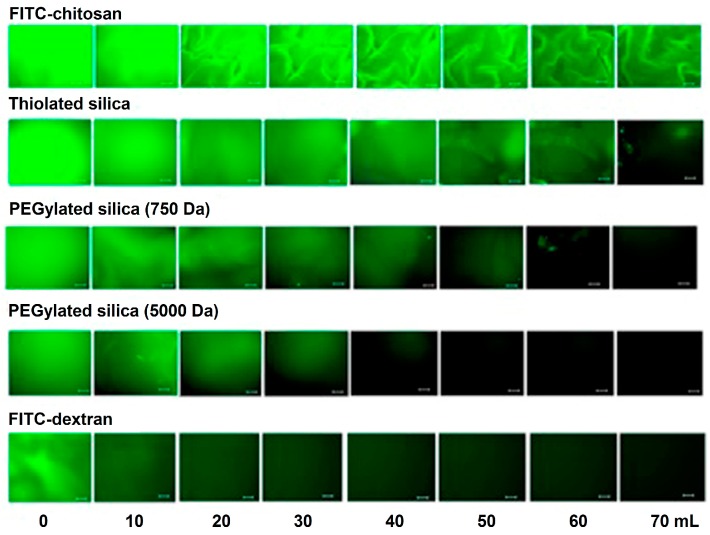
Representative microscopic fluorescence images of ex vivo porcine urinary bladder mucosa incubated with FITC-chitosan, thiolated silica, PEGylated silica (750 Da), PEGylated silica (5000 Da) and FITC-dextran and washed with different volumes of artificial urine solution. Scale bar = 200 µm. [37].

Our group has demonstrated the mucoadhesive character of chitosan in several studies. We have used a range of techniques including mucin-particle interaction [15], tensile strength [35] and most recently flow-through technique coupled with fluorescence microscopy [36,37]. In the latest case, fluorescein isothiocyanate-chitosan (FITC-chitosan) was used as a positive control and compared to other materials as well as FITC-dextran (non-mucoadhesive or negative control). Fluorescent samples were deposited onto ex vivo mucosal tissues (e.g., porcine urinary bladder or bovine eyes) and washed with bio-relevant fluids. Fluorescence images were taken after several wash cycles and the fluorescence intensity was used to compare the retention of each material on the mucosal tissues. We observed excellent mucoadhesive properties of chitosan in all cases, although some differences in the extent of its mucoadhesive potential in different mucosal tissues were noticed [37,38,39]. Figure 3 shows the result of mucoadhesion study of different silica nanoparticles in porcine urinary bladder ex vivo. The fluorescence signal of chitosan after washing was more intense compared to other materials and this indicated its excellent mucoadhesive properties. The rank of retention of materials was as follows: FITC-chitosan > thiolated silica nanoparticles > PEGylated (polyethylene glycol, 750 Da) silica nanoparticles > PEGylated (5000 Da) silica nanoparticles > FITC-dextran [37].

Behrens et al. [40] studied interactions of polystyrene, chitosan, polylactide (PLA)-PEG nanoparticles with two types of human intestinal cell lines, the enterocyte-like Caco-2 and mucus-secreting MTX-E12 cells. They revealed that the nanoparticles associated with Caco2 cells in the following order: polystyrene > chitosan > PEG-PLA. On the other hand, chitosan nanoparticles strongly bound to the mucus secreting cells and the binding of polystyrene nanoparticles was significantly decreased. PEG-PLA did not show any association with the mucus secreting cells. Intraduodenal administration of chitosan nanoparticles demonstrated that they could be internalized in both epithelial cells and Peyer’s patches. The mechanism of the transport of chitosan and polystyrene nanoparticles was studied using Caco2 cells. It was found that chitosan nanoparticles were internalized by adsorptive endocytosis, whereas non-adsorptive endocytosis could be involved with polystyrene nanoparticles. Decreasing the temperature of incubation (4 °C) significantly decreased the transport of both types of nanoparticles. Addition of 1 mM protamine sulfate (inhibitor of active transport process) and pre-treatment of the cells with 10 U/mL heparinase II or 35 mM sodium chlorate (led to de-sulfation and the removal of anionic sites of mucus and cell membranes) significantly reduced the cellular transport of chitosan nanoparticles. However, the transport of polystyrene nanoparticles did not change with these factors. Chitosan endocytosis was saturable, i.e., cellular association increased linearly with concentration (31.25–1000 µg/mL) and reached a steady state at some point. Other studies have also reported the cellular uptake enhancing effect of chitosan, which could occur by adsorptive endocytosis, where a positively-charged coated nanoparticles adhere strongly to the negatively charged components of the cell membranes [41]. 

Thongborisute et al. [42] investigated the mucoadhesion and muco-penetration of chitosan solution, liposomes and chitosan-coated liposomes in rat small intestine in ex vivo and in vivo models. The systems were fluorescently labelled with FITC and administered orally to male Wistar rats or in the ex vivo model rats were sacrificed and the samples were incubated to interact with the mucosal tissues for 1 h at 37 °C. To visualize the penetration of these materials, cross-sections of 3 different regions of the small intestine (duodenum, jejunum, and ileum) were obtained and examined with confocal laser scanning microscopy (CLSM). They showed that chitosan, non-coated liposomes, and chitosan-coated liposomes could adhere and penetrate the mucosal tissues. However, the extent of adhesion and penetration of chitosan-coated liposomes was greater than for non-coated liposomes. The authors related this behavior to firstly, the mucoadhesive properties of chitosan. Secondly, the presence of chitosan on the surface of liposomes could result in the formation of large aggregates due to the interactions of chitosan macromolecules leading to the large network of chitosan-coated liposomes adhering to the mucus layer. This phenomenon is not observed in the case of non-coated liposomes and only individual particles disperse in the suspension. Interestingly, although the authors did not discriminate the mucoadhesion and the mucosal penetration, they observed more mucosal penetration in the ileum region compared to both duodenum and jejunum, which they believe was due to the thicker nature of the ileum, which is also supported by other studies ([43,44,45]. Deacona et al. [46] also revealed the difference in the mucoadhesive interactions of chitosan in different regions of porcine stomach by sedimentation velocity technique using analytical ultracentrifuge equipped with conventional Philpott-Svensson Schlieren optical systems and coupled on-line to a charge-coupled device (CCD) camera. The cardiac region displayed the strongest interaction with chitosan compared to corpus and antrum. 

## 3. Problems of Chitosan in Mucosal Drug Delivery

Being a basic polymer, chitosan is mucoadhesive only at limited pHs and is only soluble at acidic pH (pH < 6) [17,47]. The requirement of decreasing the pH of chitosan vehicles limits its applications in drug and gene delivery as many biomolecules including DNA, proteins and peptide-based drugs are not stable at low pH [48]. Additionally, even acidic chitosan formulations will encounter neutral to basic pHs once they administered into the human body either topically or systemically. High pH environment results in the precipitation of chitosan and can affect the performance of the carrier systems [17].

Chitosan-based mucosal drug delivery systems have been investigated to increase the residence time of drugs on the application/absorption sites [30,33,35]. The increase in the residence time is advantageous as it may prolong the action of drugs and provides sustained drug release. However, with unmodified chitosan, this is only possible to a certain degree. Therefore, there is an obvious need for further controlled drug release with subsequent prolongation of drug action [49].

Several modifications of chitosan have been investigated to enhance its mucoadhesive properties. In the next sections, we will discuss various chitosan derivatives with potential applications in transmucosal drug delivery.

## 4. Mucoadhesive Chitosan Derivatives

### 4.1. Trimethyl Chitosan (TMC)

**Figure 4:**
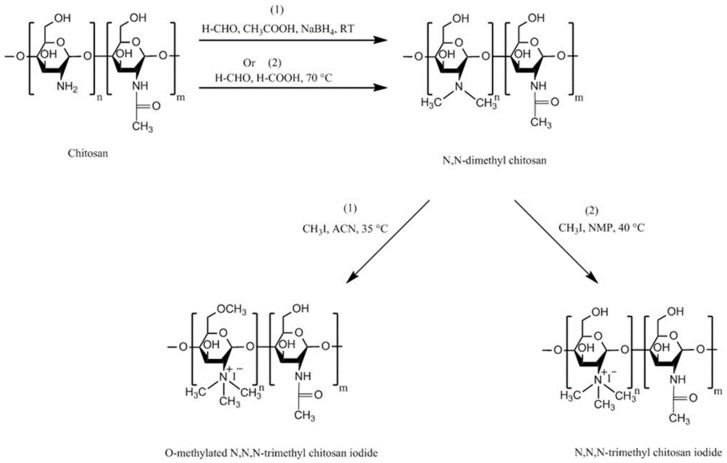
Synthetic pathway for preparation of TMC using indirect trimethylation approach according to (1) Muzzarelli and Tanfani [50], ACN = acetonitrile and (2) Verheul et al. [51] avoiding *O*-methylation, NMP = *N*-methyl-2-pyrrolidinone.

**Figure 5:**
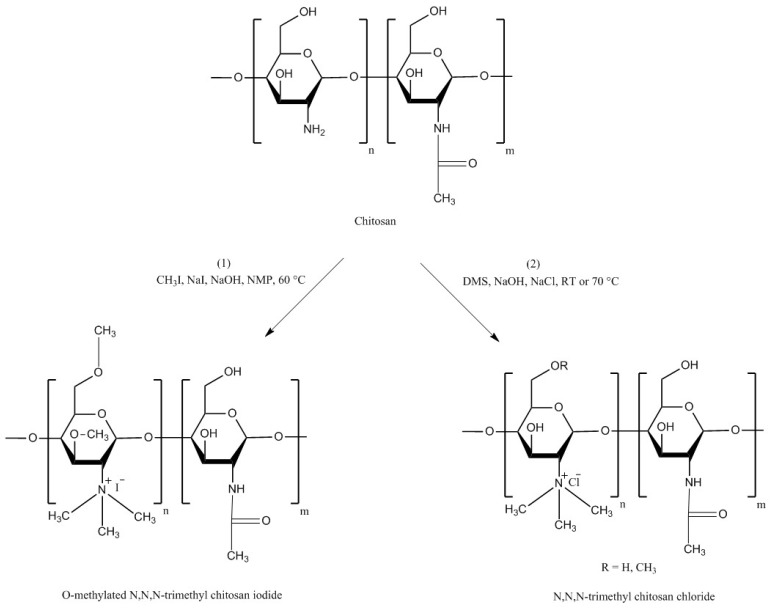
Synthetic pathway for preparation of TMC using direct trimethylation approach according to (1) Sieval et al. [52], NMP = *N*-methyl-2-pyrrolidinone and (2) de Britto and Assis [53], DMS = dimethyl sulfate.

**Figure 6:**
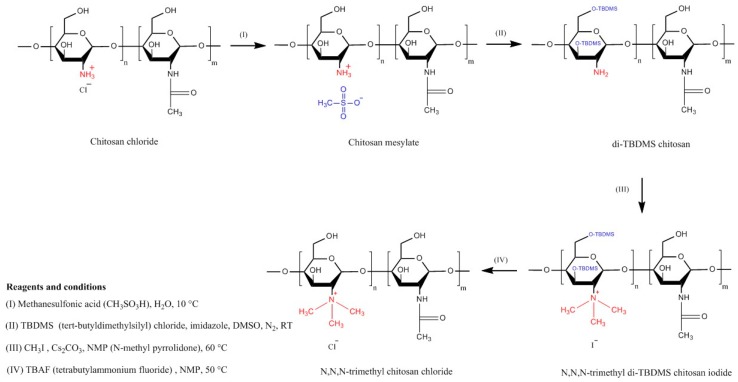
Synthetic pathway for preparation of TMC using hydroxyl groups protection approach by *O*-silylation according to Benediktsdóttir et al. [54].

TMC is a chitosan derivative which is always positively charged. This persistent cationic nature makes it one of the strongest mucoadhesive polymers. It has a much wider pH solubility range than unmodified chitosan due the presence of protonated groups (–N^+^(CH_3_)_3_) [16]. TMC can be synthesized by three general methods: indirect trimethylation [50,51], direct trimethylation [52,53] and protection of chitosan hydroxyl groups (at C-3 and C-6 positions) by *O*-silylation [54]. The first method is usually a two-step process including the formation of an intermediate product (*N*,*N*-dimethyl chitosan) and can be conducted using two different reaction conditions. Whereas, the second method is a one-step process and does not contain any intermediate product, but it can also be conducted using two different reaction conditions. Using either indirect or direct trimethylation can often result in the formation of *O*-methylated TMC. However, using hydroxyl protection method by *O*-silylation, e.g., by employing tert-butyldimethylsilyl chloride, *O*-methylation can be avoided [16,50,51,52,53,54,55]. Verheul et al. [51] also claimed that their synthetic approach can result in *O*-methyl free TMC. The synthetic pathway for each method is illustrated in Figure 4, Figure 5 and Figure 6. For the details of the experimental methods of TMC synthesis, readers are referred to two recent reviews by Wu et al. [55] and Kulkarni et al. [16].

TMC has been synthesized so as to enhance the water-solubility of chitosan with wider applications in drug delivery [56]. Subsequently, Sieval et al. [52] studied the effect of a few variables including the number of reaction steps, the duration of each reaction step and the amount of methyl iodide as a reagent. It was found that 2-step reaction resulted in products with high degree of substitution (40–80%). However, 3-step reaction led to even greater degree of substitution but at the same time water-solubility of the resulting product decreased. 

Jintapattanakit et al. [57] synthesized TMC by reductive methylation of chitosan. TMC was then PEGylated. Both polymers were then fluorescently labelled using tetramethyl-rhodamine isothiocyanate (TRITC) and Oregon Green carboxylic acid succinimidyl ester (Oregon Green 448). The insulin-loaded nanoparticles were synthesized using self-assembly technique. The influence of TMC PEGylation and its positive charge density on mucoadhesive properties were assessed using a mucin assay and mucus-secreting HT29-MTX-E12 (E12) monolayers. It was found that introduction of PEG improved the mucoadhesive effect of TMC. This could be due to the interpenetration of PEG with mucus. In some other studies, PEGylation of chitosan also shown reduced toxicity and significantly increased the cellular permeation of hydrophilic macromolecules including FITC-dextran [23,58].

Hauptstein et al. [59] also studied the effect of PEGylation as well as thiolation (will be discussed in the next section) on adhesion of chitosan’s compressed discs to porcine intestinal mucosa. They synthesized PEG-bearing thiolated chitosan by conjugating thiol-bearing polyoxyethylene ligand [*O*-(3-carboxylpropyl)-*O*′-[2-[3-mercaptopropionylamino)ethyl]-polyethyleneglycol] to amino groups of chitosan. The reaction was mediated by 1-ethyl-3-(3-dimethylaminopropyl)-carbodiimide hydrochloride (EDAC)/*N*-hydroxysuccinimide (NHS). In addition to its solubility in basic media, PEG-bearing thiolated chitosan showed greater mucoadhesive strength compared to unmodified chitosan. However, it was equally mucoadhesive as thiolated chitosan. Moreover, PEG-bearing thiolated chitosan enhanced the permeation of FITC-dextran through rat intestinal mucosa and Caco2 cells monolayer. The enhancement in mucoadhesion is based on the formation of disulfide bridges with mucus glycoproteins. The permeation enhancing effect could be due to the interaction of thiol groups of the thiolated chitosans with protein tyrosine phosphatase enzyme, which modulates the tight junction by a glutathione-dependent process.

Sayın et al. [60] demonstrated a novel approach for formation of nanoparticles via complexation between cationic TMC and polyampholytic *N*-carboxymethylchitosan without a crosslinker. The nanoparticles were loaded with FITC-BSA (bovine serum albumin) and their cellular uptake was studied. A significant number of the nanoparticles was taken up by murine macrophage J774A.1 within 30 min of incubation. The authors believed that the mucoadhesive effect of TMC plays a major role in the enhancement of the cellular uptake. The nasal administration of tetanium toxiod-loaded 283 nm nanoparticles in mice, induced the mucosal and systemic immune responses. 

Sajomsang et al. [61] synthesized two methylated *N*-aryl chitosan derivatives, methylated *N*-(4-*N*,*N*-dimethylaminocinnamyl) chitosan chloride and methylated *N*-(4-pyridylmethyl) chitosan chloride by reductive amination and methylation of chitosan. It was found that increasing the degree of quaternization led to a stronger mucin-particle interaction. Moreover, the cytotoxicity was dependent on the polymer structure, the location of the positive charge and the molecular weight after methylation.

On the other hand, some studies showed that TMC has greater potential to adhere to the epithelial tissue than to the mucin. For instance, Keely et al. [62] evaluated the adhesion of coumarin-labelled-poly(2-dimethylaminoethyl) methacrylate (pDMAEMA) with different levels of quaternization (0, 10, 24 and 32%) and TMC to human mucus-secreting and non-mucus-secreting intestinal cell monolayers (E12 and HT29, respectively) as well as freshly excised rat intestinal mucosa using non-everted intestinal sacs model. CLSM, light and fluorescence microscopy were used to quantify either mucoadhesion (adhesion to the mucus layer) or bioadhesion (adhesion to the epithelial tissue rather than mucosal surface). It was found that pDMAEMA, regardless of the degree of quaternization, was more mucoadhesive than bioadhesive, whereas TMC was found to be more bioadhesive and as mucoadhesive as unquaternized pDMAEMA and 24% quaternized pDMAEMA. When E12 cells and intestinal sacs were treated with mucolytic agent, *N*-acetylcysteine, for 15 min, the mucoadhesion of pDMAEMA polymers was significantly decreased, while the bioadhesion of TMC had not changed following this treatment. Additionally, the permeability of FITC-dextran through both E12 cells monolayer and intestinal sacs was significantly decreased in the presence of pDMAEMA, whereas the use of TMC led to a significant increase in the permeability. Although they did not study the interactions between the polymers and the mucus, the authors claimed that pDMAEMA perhaps increased the viscosity of the mucus gel as in case of carbopol [63] and thus impede the diffusion of FITC-dextran. However, chitosan and its derivatives can open the tight junctions [64,65,66] that could enhance the paracellular diffusion of FITC-dextran.

Liu et al. [67] developed core-shell nanoparticles based on TMC. The nanoparticles were coated with dissociable layer of *N*-(2-hydroxypropyl) methacrylamide copolymer (pHPMA). The diffusion of coated and uncoated nanoparticles in human cervicovaginal mucus was evaluated using multiple particle tracking technique and Ussing chamber. Cellular internalization and transport were evaluated using E12 cells. It was found that pHPMA coating could enhance the diffusion of TMC nanoparticles through both mucus and epithelial layer. Non-coated TMC nanoparticles were found to be less diffusive in both mucus and the cells. Liu et al. [67] indirectly demonstrated the mucoadhesive properties of TMC. 

Generally, mucoadhesive properties of chitosan could be affected by both the degree of quaternization and its molecular weight. Nazar et al. [68] prepared TMC thermosensitive nasal gel from low, medium, and high molecular weight chitosan with quaternization of 25.6 to 61.3%. It was found that gels made from lower quaternization and medium molecular weight TMC had the greatest work of adhesion (252 ± 14 μJ) and the shortest sol-gel transition time (7 min) at 32.5 °C. This could be due to their great capacity to hydrate and absorb large amounts of water. Partially quaternized TMC has the advantage of having a better water solubility profile in neutral and basic environment than the native chitosan [69]. This is important since absorption of most drugs happens at slightly basic or neutral part of the gastrointestinal tract [70]. 

TMC has been used as an absorption enhancer for the delivery of buserelin and insulin across Caco-2 cells monolayers. Although at low concentrations TMC is a less active absorption enhancer than both chitosan hydrochloride and chitosan glutamate, increasing its concentration could increase its activity. Since it is more soluble than both chitosan salts, increasing TMC concentration is very unlikely to cause precipitation, however, it resulted in an increase in the transport rate of both buserelin and insulin across Caco-2 cell monolayers, which might be due to the decrease in transepithelial electrical resistance (TEER) [71]. TEER is a parameter, which determines the intercellular ion flux and indicates the tightness of paracellular “junctional complexes” of biological membranes [72].

### 4.2. Carboxymethyl Chitosans

Carboxymethyl chitosan is another derivative of chitosan with amphoteric properties, acting as both acid and base depending on the pH of its solution. The amphoteric properties originate from the presence of both amino (basic) and carboxylic (acidic) groups in its chemical structure [73,74,75]. The amino groups undergo protonation in acidic media and make carboxymethyl chitosan positively charged. On the other hand, in basic media carboxylic groups dissociate and impart carboxymethyl chitosan negative charged. 

Chen and Park [76] studied the pH-solubility profile of various *O*-carboxymethyl chitosans synthesized at different reaction conditions (temperature and ratio of water/isopropanol). The resultant chitosans showed a pH-dependent water-solubility character. Based on the degree of substitution, carboxymethyl chitosans (0.2 mg/mL) were insoluble at pH ranges close to neutral. However, at highly acidic and basic pHs, they demonstrated complete water-solubility. It was found that using low temperature (0 and 10 °C, during the synthesis) resulted in completely water-soluble products but with low yield. Increasing the temperature and decreasing the water/isopropanol ratio resulted in more carboxymethylation, which subsequently shifted the region of insolubility towards the lower pH (~3). Vikhoreva and Gal’braikh [77] also reported that carboxymethyl chitosan was insoluble at pH range of 3.5–6.5, whereas it showed complete solubility at pH < 3.5 and > 6.5. The insolubility at those pH ranges could be due to the fact that the isoelectric point of carboxymethyl chitosan is 4.1 and therefore when the pH of the solution is near the isoelectric point, precipitation and aggregation could happen [73].

**Figure 7:**
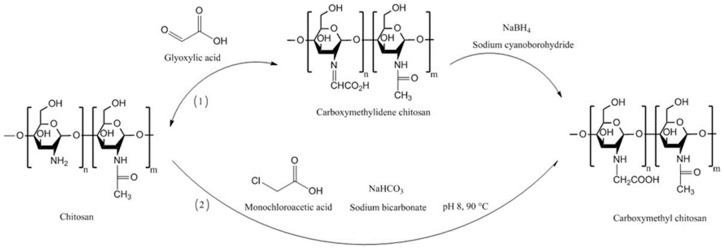
Schematic representation of the synthesis of carboxymethyl chitosans using reductive (1) [73] and direct (2) alkylation [79] methods.

Generally, carboxymethyl chitosans can be prepared using two different approaches, which are reductive alkylation and direct alkylation. In case of reductive alkylation, the amino groups of chitosan react with aldehyde groups of glyoxylic acid to form an intermediate imine product, which then is hydrogenated using sodium borohydride or sodium cyanoborohydride. The ratio of glyoxylic acid to chitosan is important in determining whether mono- or di-carboxymethyl chitosan is formed. Direct alkylation can be performed by reacting chitosan with some alkyl halides, such as monochloroacetic acid, in the presence of inorganic bases including sodium bicarbonate and sodium carbonate to raise the pH to 8.0–8.5. The pH of the reaction mixture is considered to be one of the important factors in determining whether *O*-, *N*- or *O*, *N*-substitution takes place [74,78,79,80]. Also, the higher pH resulted in a greater degree of substitution [81]. Figure 7 shows the pathways for the synthesis of carboxymethyl chitosans.

Di Colo et al. [82] studied the effect of chitosan and *N*-carboxymethyl chitosan on the ocular pharmacokinetics of ofloxacin. Chitosan enhanced the penetration of the drug through the ocular tissue and its maximum concentration (C_max_) in the aqueous humor was greater than in the case when conventional eye drops (Exocin^®^ eye drops) and reference formulation (polyvinyl alcohol-based ofloxacin solution) were used. This may be due to the tight junction opening effect of chitosan. *N*-carboxymethyl chitosan did not significantly enhance the C_max_ of the drug in the aqueous humor. However, it resulted in a steady state drug concentration from 30–150 min post-ocular administration. The authors measured the viscosity of the three formulations and found that they were approximately similar. However, they still claimed that the viscosity enhancement is one of the reasons for the enhancement of pre-ocular drug residence time compared to the reference formulation. The binding of ofloxacin to *N*-carboxymethyl chitosan due to hydrogen bonding between amino groups of the drug and hydroxyl groups of the polymer, is also a reason for both the decrease in the ocular drug penetration and the increase in the residence time [82]. Although they did not evaluate the mucoadhesive properties of these polymers, they hypothesized that it could have an impact on the increased residence time in the ocular tissues. Clearly, the residence time of a formulation on the ocular tissues will be related to their mucoadhesive properties.

*N*-carboxymethyl chitosan has also been used as an intestinal absorption enhancer and proved to increase the in vitro and in vivo transmucosal absorption of low molecular weight heparin [73]. It has also showed potential in the oral delivery of small molecules. Prabaharan and Gong [83] synthesized thiolated carboxymethyl chitosan-*g*-β-cyclodextrin and showed its potential for the oral delivery of lipophilic drug ketoprofen. The modified chitosan resulted in 5-fold improvement in the adhesion to rat intestinal mucosa and slower drug release.

### 4.3. Thiolated Chitosans

**Figure 8:**
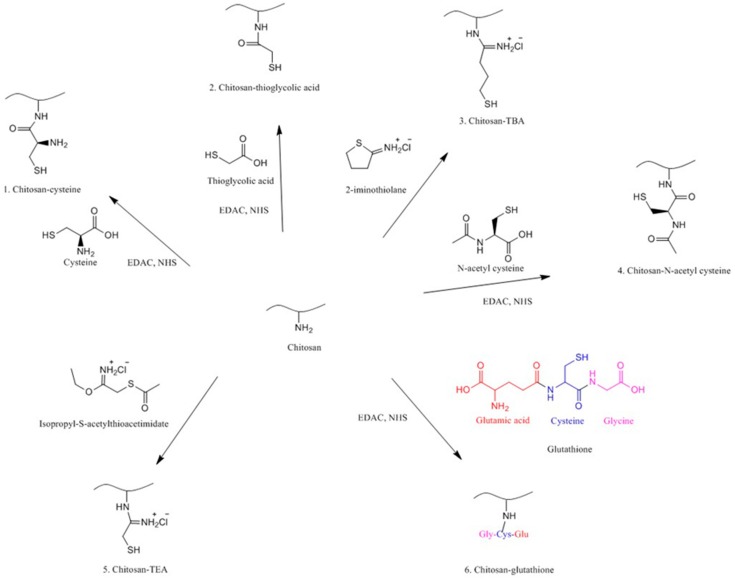
Synthetic pathways to different thiolated chitosan derivatives [14,84,85,86,87,88,89,91].

Thiolation is one of the techniques used to functionalize various polymers including chitosan using thiolating agents bearing thiol groups. These include cysteine [84], thioglycolic acid (TGA) [85], 2-iminothiolane or 4-thiobutylamidine (TBA) [86], *N*-acetyl cysteine [87], isopropyl-S-acetylthioacetimidate [88] and glutathione [89]. This technique has been pioneered by Bernkop-Schnürch and co-workers [90] to enhance the mucoadhesion of polymers for pharmaceutical and biomedical applications. Thiolated chitosans are now one of the extensively studied mucoadhesive materials. Despite their superior mucoadhesive properties, they also have some permeation enhancing effects, ability to inhibit efflux pumps and in situ gelling properties [25]. Figure 8 shows the synthetic pathways to different thiolated chitosans.

#### 4.3.1. Chitosan-Cysteine

In 1999, Bernkop-Schnürch et al. [84] synthesized chitosan-cysteine conjugate by covalent attachment of cysteine to chitosan mediated by carbodiimide, where the amount of bound-cysteine was increased with an increase in the amount of the mediator reaching 1.2%. Subsequent mucoadhesion study revealed no significant difference between chitosan and thiolated chitosan. However, thiolated chitosan tablets showed superior cohesion over the chitosan tablets which could be due to the formation of intra/intermolecular disulfide bonds as a result of the oxidation of the thiol groups in thiolated chitosan. This improved cohesion is desirable not only for the mucoadhesion but also for the design of controlled release dosage forms [14,84].

TMC has also been thiolated by reacting with cysteine mediated with EDAC/*N*-NHS. Insulin-loaded nanoparticles were prepared using polyelectrolyte complexation method. The resultant TMC-cysteine showed significantly greater mucoadhesion capacity compared to unmodified TMC in both rat ileal loop and mucin adsorption models. This might be due firstly to the electrostatic interaction between positively charged chitosan and negatively charged sialic acid of mucin glycoproteins leads to the interpenetration of the polymer and mucin. Secondly, at neutral pH (pH of small intestine) the thiol groups of TMC-cysteine could be oxidized by reacting with cysteine-rich domains of mucin leads to the formation of disulfide bonds, which finally may immobilize more thiolated polymeric particles in the mucus layer than the unmodified polymer [92]. TMC-cysteine nanoparticles also showed greater permeability enhancement effect compared to unmodified TMC, which can be linked to the inhibition of protein tyrosine phosphatase which facilitates opening of tight junctions [14]. It might also be due to the greater mucoadhesion of TMC-cysteine than the native chitosan. Third possible reason is the inhibition of protease activities on insulin via shielding of enzymatic cutting sites after formation of self-assembled nanoparticles [92].

#### 4.3.2. Chitosan-*N*-Acetyl-Cysteine

Schmitz et al. [87] synthesized chitosan-*N*-acetyl-cysteine conjugate via covalent attachment of *N*-acetyl-cysteine to chitosan using two different concentrations of EDAC as a mediator. They observed that this modification resulted in 50-fold increase in the retention of chitosan compressed discs on ex vivo porcine intestinal mucosa. The total work of adhesion required to detach the chitosan-*N*-acetyl-cysteine discs from the intestinal mucosa was 8.3-fold greater than unmodified chitosan. This may be due to the increase in the number of disulfide bonds between the polymers and the cysteine-rich domains of mucosa. They also revealed that increasing the concentration of EDAC resulted in products with greater amount of thiol groups. This is due to the activation of carboxylic groups of *N*-acetyl-cysteine, which resulted in immobilization of more thiol groups on the polymer. This eventually increased its mucoadhesive strength.

#### 4.3.3. Chitosan-Thioglycolic Acid (Chitosan-TGA)

Chitosan-TGA has been synthesized by introducing TGA to chitosan using EDAC as a mediator. The resulting thiolated chitosan showed 4.3-fold increase in the viscosity, which is desirable for mucosal drug and gene delivery and scaffold materials in tissue engineering. This improvement in the viscosity may be related to the formation of disulfide bonds within the polymeric matrix [85]. The viscosity of this thiolated chitosan can be further improved by using different oxidizing agents including hydrogen peroxide, sodium periodate, ammonium persulfate and sodium hypochlorite. These agents accelerated the sol-gel transition to take place only within few min, while without them this transition requires 40 min. 25 nmol/L hydrogen peroxide has increased the dynamic viscosity of 1% chitosan-TGA solution by up to 16,500-fold. This may be due to the formation of more inter- and intra-chain disulfide bonds [93]. To assess the potential of chitosan-TGA for non-viral oral gene delivery, 100–200 nm nanoparticles with zeta potential of 5–6 mV have been formed by complex coacervation of plasmid DNA and the thiolated chitosan. These particles showed acceptable stability toward DNase and thus resulted in a 5-fold increase in the rate of transfection [94] .

In another study, Barthelmes et al. [95] synthesized mucoadhesive nanoparticles based on chitosan-TGA using ionic gelation with sodium tripolyphosphate (TPP) for intravesical drug delivery. Two types of partially oxidized (different in their disulfide content, -SH groups oxidized to form -S–S- bonds) chitosan-TGA-TPP nanoparticles were also synthesized by the addition of H_2_O_2_ solution (0.5% *v*/*v*) to chitosan-TGA-TPP nanoparticles. Either fluorescein diacetate or trimethoprim were then loaded into the nanoparticles. Then, using a flow through technique, the amount of fluorescein diacetate adhered to the bladder mucosa was quantified using fluorescence spectrophotometry. It was found that using chitosan-TGA-TPP nanoparticles, 14.2 ± 7.2% of fluorescein diacetate remained on the surface of the mucosal tissues but in the case of unmodified chitosan-TPP nanoparticles, only 1.1 ± 0.1% fluorescein diacetate remained after washing with simulated artificial urine for 3 h with a flow rate of 2 mL/min. This improvement in the mucoadhesion was due to the covalent bonds formed between the thiol groups of the polymers and the cysteine-rich domains of the glycosaminoglycan layer of the mucus which is composed of proteoglycans and glycoproteins as in the case of adhesion to the intestinal mucosa [95,96]. To prove the concept, a quantitative analysis of free thiol groups of intestinal and urinary bladder mucus was performed and revealed no significant difference between the thiol contents of the two mucosal tissues. Interestingly, release study using artificial urine as a dissolution media shown that covalently crosslinked chitosan-TGA-TPP nanoparticles resulted in a slower and more controlled release of trimethoprim compared to ionically crosslinked chitosan-TGA-TPP and unmodified chitosan-TPP nanoparticles. The nanoparticles with greater content of disulfide bonds released the drug significantly slower than the nanoparticles with fewer disulfide bonds. The authors suggested that covalent crosslinking resulted in harder nanoparticles due to the formation of disulfide bridges within the matrix of the nanoparticles. This then increased the mechanical strength of the nanoparticles and thus made the artificial urine diffuse slowly into the nanoparticles. Consequently the dissolution of trimethoprim decreased and the nanoparticles released the drug slowly [95].

#### 4.3.4. Chitosan-4-Thiobutylamidine

Chitosan-4-thiobutylamidine (chitosan-TBA) is another type of thiolated chitosan with mucoadhesive properties [97]. It remained on porcine small intestinal mucosa for 161 ± 7 h when tested using rotating cylinder method. In addition, the total work of adhesion was 740 ± 147 µJ. It has been reported that the mucoadhesive property of thiolated chitosans is pH dependent, and this point should be considered in the design of thiolated chitosan-based mucosal drug delivery systems [97].

Langoth et al. [98] designed mucoadhesive buccal delivery system of pituitary adenylate cyclase-activating polypeptide using chitosan-TBA as a promising treatment for type-2 diabetes mellitus. The in vivo buccal administration through porcine buccal mucosa resulted in a continuous rise in the plasma level of the enzyme over 6 h. 

**Figure 9:**
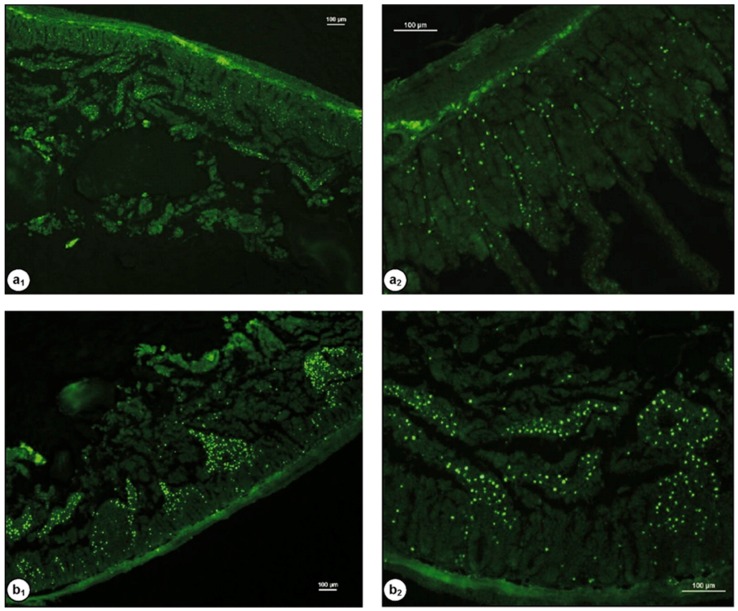
Fluorescent images of rat intestinal tissues after 2 h incubation with 100 µL (0.5% *w*/*v*) chitosan (**a**) and chitosan-TBA (**b**) nanoparticles labelled with Alexa Fluor 488, (a_1_ and b_1_, 40×; a_2_ and b_2_, 100× magnification). The scale bars = 100 µm. Reprinted from [99] with permission of Elsevier.

Dünnhaupt et al. [99] synthesized fluorescently-labelled nanoparticles of chitosan-TBA and polyacrylic acid-cysteine conjugate using ionotropic gelation technique. For the mucoadhesion study, fresh jejunum of rats was cut into 2 cm segments and filled with 0.1 mL nanoparticles. After fixation, the mucosal tissues were examined by fluorescence microscopy. The penetration study was performed using fresh “mucus-filled silicon tube” technique. It was found that nanoparticles of both modified chitosan (Figure 9) and polyacrylic acid exhibit greater mucoadhesive strength than unmodified nanoparticles. Chitosan particles showed 2-fold greater mucoadhesive property than polyacrylic acid particles. On the contrary, the muco-penetration ability of unmodified nanoparticles was greater than the thiolated nanoparticles.

The combination of chitosan-TBA and chitosan-Bowman-Birk inhibitor in the design of 2 mg enteric coated microtablets showed a significant enhancement in the effect of oral salmon calcitonin on the level of plasma calcium when tested in rats [100]. The derivatization of chitosan with enzyme inhibitors will be discussed in a separate section.

#### 4.3.5. Chitosan-Thioethylamidine

The use of 2-iminothiolane to synthesize thiolated chitosan resulted in a marked increase in the mucoadhesion. However, the resultant thiolated chitosan lacks sufficient stability leading to the reduction in the number of free thiol groups. One of the reasons for the instability could be the formation of *N*-chitosanyl-substituted 2-iminothiolane structures, which happens after modification of some amines using 2-iminothiolane. This intermediate product loses ammonia and results in the formation of re-cyclized *N*-substituted 2-iminothiolanes.To avoid this side reaction, Kafedjiiski et al. [88] synthesized thiolated chitosan using isopropyl-S-acetylthioacetimidate as a thiolating agent and an alternative to 2-iminothiolane. In contrast to chitosan-TBA (higher than unmodified chitosan) [101], the swelling property of chitosan-thioethylamidine was not significantly different from unmodified chitosan. However, the mucoadhesion was significantly improved. Using chitosan-thioethylamidine, the release of FITC-dextran was sustained over 3 h, which could be due to the presence of disulfide bonds in the structure of chitosan, which can slow the diffusion of FITC-dextran macromolecules down.

#### 4.3.6. Chitosan-Glutathione

Several studies reported the use of glutathione for the synthesis of chitosan-glutathione conjugates [89,91,102,103]. Due to its permeation-enhancing effect, redox potential and safe toxicological profile, glutathione is a suitable thiolating agent for biomedical applications. Due to the presence of thiol groups in the glycine part of glutathione, it has strong electron donating property, acting as a reducing agent. Additionally, the stability of glutathione against cellular aminopeptidase is provided by the presence of ɤ-peptidic bond between glutamic acid and cysteine. Also, its conformational flexibility, makes glutathione a highly reactive ligand [89]. 

Similar to other thiolated chitosans, the synthetic approach is based on the formation of amide bonds between glycine carboxylic acid groups of glutathione and amino groups of chitosan. The reaction can be mediated by EDAC/NHS. The method was developed by Kafedjiiski et al. [89]. The resultant chitosan-glutathione exhibited acceptable cohesive properties and did not disintegrate in physiological solution (0.1 M phosphate buffer solution pH 6.8) for 48 h. However, unmodified chitosan was only stable for 9 h. Interestingly, both polymers showed the same swelling behavior, whereas chitosan glutathione had greater mucoadhesive properties (expressed as the total work of adhesion and tablets-intestinal detachment time) than unmodified chitosan. The apparent permeability of rhodamine 123 using chitosan-glutathione and unmodified chitosan were 2.06 × 10^−7^, and 0.66 × 10^−7^ cm/s, respectively.

Jin et al. [104] demonstrated the application of chitosan-glutathione in the oral delivery of thymopentin (a synthetic pentapeptide with immune-regulatory action). They synthesized thymopentin-loaded poly(butyl cyanoacrylate) nanoparticles using emulsion polymerization technique. The particles were subsequently coated with either chitosan or chitosan-glutathione and orally administered to immunosuppressed rats. It was found that chitosan-glutathione-coated nanoparticles were able to normalize the immune function of rats, which is probably due to the enhanced mucoadhesive properties of chitosan-glutathione.

Chitosan-glutathione hydrogel was also found to be more effective in the reduction of oxidative stress in neonatal rat cardiomyocytes than unmodified chitosan hydrogel. The action possibly related to better cellular adhesion potential of chitosan-glutathione compared to unmodified chitosan as a result of the availability of the biocompatible glutathione promoting the cells survival [91].

#### 4.3.7. Comparison of Chitosan, Trimethyl Chitosan and Thiolated Chitosan

In a comparative study, Mei et al. [105] investigated the mucoadhesion as well as the nasal absorption enhancing effect of chitosan, thiolated chitosan and trimethyl chitosan. Chitosans of different molecular weights were synthesized by depolymerization then the depolymerized samples were either trimethylated as reported in [106] or thiolated by reacting with cysteine using EDAC/NHS chemistry according to Bernkop-Schnürch and Steininger [107] with slight modification. The mucoadhesion of chitosan and thiolated chitosan was evaluated and the detachment time of 5 mm discs of the polymers from freshly excised porcine intestinal mucosa was evaluated. Discs of thiolated chitosan with greater degree of substitution (152 µmol/g) detached in a significantly longer time (about 12 h) than unmodified chitosan. The bioavailability of 2,3,5,6-tetramethylpyrazine phosphate through nasal route after its formulation with different chitosans was investigated. It was found that the use of any type of chitosan (unmodified, thiolated and trimethyl chitosan) resulted in a significantly improved absorption of 2,3,5,6-tetramethylpyrazine, however, no significant difference between thiolated chitosans (two different degrees of substitution) with unmodified chitosan was observed. The authors claimed that the permeation-enhancing effect is dose- and molecular weight-dependent and 100 kDa resulted in maximal absorption enhancement. On the other hand, trimethyl chitosan led to a significant enhancement in the nasal absorption of the drug. These results contradict those studies reporting the absorption enhancing effect of thiolated chitosan through intestinal mucosa. For example, Krauland et al. [101,108] demonstrated that chitosan-4-thiobulyamidine resulted in an increase in the oral and nasal absorption of insulin compared to unmodified chitosan. In Krauland et al. studies [101,108], the absorption enhancement could also be due to the inhibition of protein tyrosinase and P-glycoprotein efflux pump in the mucosal membranes [101,109].

#### 4.3.8. Pre-Activated (S-Protected) Thiolated Chitosans

Vulnerability of thiolated chitosans to oxidation can be considered as one of the major limitations of their use as mucoadhesive polymers. Thiolated chitosans are generally stable in dry state. However, in solutions, they undergo rapid oxidation especially in the presence of oxidants such as oxygen and particularly at pH ˃ 5 [86]. This, will not only lead to the formation of intra- and inter-molecular disulfide bonds, but also results in the reduction of the free thiol groups necessary for the formation of disulfide bridges with the cysteine-rich domains of the mucin. This will then lead to a significant reduction in the mucoadhesive potential of thiolated chitosans under physiological conditions of the gastrointestinal tract [86]. To prevent the unwanted oxidation of thiolated chitosans, pre-activated or S-protected thiolated chitosans have been developed by Bernkop-Schnürch and co-workers. 

Generally, pre-activated thiolated chitosan can be synthesized by two steps. Firstly, thiolated chitosan is prepared using a thiolating agent and secondly thiol groups are protected by disulfide bond formation using ligands with mercaptopyridine substructure including mercaptonicotinamide, mercaptonicotinic acid and mercaptopyridine. Due to its toxicity profile mercaptopyridine is less commonly used [25]. Despite improvement of mucoadhesive properties, S-protection can also enhance the intestinal permeability of hydrophilic molecules such as FITC-dextran. In addition, S-protected thiolated chitosans have shown less cellular toxicity than the unprotected chitosans [110]. 

**Figure 10:**
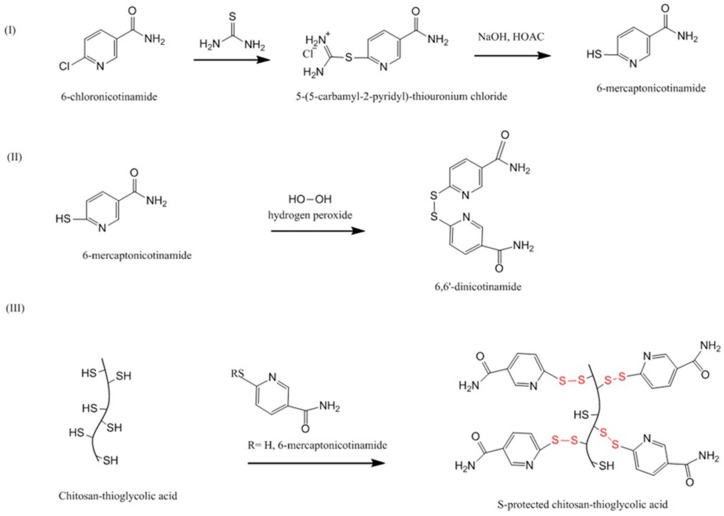
Synthetic pathway to S-protected chitosan-thioglycolic acid [111].

Dünnhaupt et al. [111] synthesized S-protected thiolated chitosan using a two-steps approach (Figure 10). First, thioglycolic acid was covalently attached to chitosan and resulted in the formation of amide bonds between the amino groups of chitosan and the carboxylic groups of thioglycolic acid. Secondly, aromatic ligand 6-mercaptonicotinamide (6-MNA) was synthesized by reacting 6-chloro-nicotinamide with thiourea, which was then oxidized using hydrogen peroxide to form 6, 6′-dithionicotinamide (6, 6′-DTNA). Both 6-MNA and 6, 6′-DTNA were then reacted with thiolated chitosan to obtain S-protected thiolated chitosan. Tablets of unmodified, thiolated and S-protected thiolated chitosans were prepared. Using rotating cylinder method, it was found that S-protected thiolated chitosan with 660 µmol/g thiol groups remained attached to the intestinal mucosa for 90 h, whereas unprotected thiolated chitosan were only attached for 45 h. However, it seemed there was no significant difference between unprotected and S-protected thiolated chitosan with more thiol groups (980 µmol/g). Unmodified chitosan detached after only 10 h. Rheological studies also indicated that mixing S-protected thiolated chitosan with mucin resulted in a significant increase in the apparent viscosity of the mixture compared to both unmodified and unprotected thiolated chitosan. The authors believed that S-protected thiolated chitosan interacts more rapidly and quantitatively with mucus by thiol-disulfide exchange reaction between the thiol groups of mucus-cysteine and the pyridyl-thiol moiety of the S-protected thiolated chitosan. In the mucus, the amount of free thiol groups (-SH) is approximately two times greater than their oxidized form (-S–S-) [95] and this is in favor of thiol-disulfide exchange. Thus, more bonding between S-protected thiolated chitosan and the mucus can be achieved compared to unprotected thiolated chitosan [111].

In another study, Dünnhaupt et al. [112] demonstrated the application of S-protected chitosan-TGA (chitosan-TGA-MNA) in the oral delivery of antide as tablets dosages forms. It was shown that hardness of chitosan-TGA-MNA tablets was significantly increased due to introduction of 6-MNA ligand and the presence of disulfide bonds within the polymeric network. Chitosan tablets swelled quickly and reached maximum within 2 h. However, chitosan-TGA tablets swelled slowly and continuously with greater extent than the unmodified chitosan. The presence of disulfide bonds might explain the enhanced water absorbing capacity of chitosan-TGA. On the other hand, chitosan-TGA-MNA tablets swelled to a lesser extent (1.5-fold) than chitosan-TGA tablets, which could be due to the presence of hydrophobic 6-MNA ligand. Additionally, chitosan-TGA-MNA resulted in a constant sustained release of antide and after 8 h, only 65% released. However, the% of antide released from chitosan-TGA and unmodified chitosan were 77 and 100%, respectively. The in vivo study in male Sprague Dawley rats, however, indicated only a slightly higher plasma concentration of antide, but not statistically significant (*p* > 0.05) using chitosan-TGA-MNA compared to chitosan-TGA. The authors claimed that this compromise in the oral bioavailability of antide could be due to the enhanced cohesiveness and controlled release of chitosan-TGA-MNA tablets. These two properties are essentially important in the design of mucoadhesive formulations as if the polymer is not cohesive enough it might collapse and therefore the peptide might not be protected and rapidly released into the lumen of the gastrointestinal tract and degraded and no longer contributes to the concentration gradient [112].

#### 4.3.9. Other Thiolated Chitosans

Thiolated methylated dimethylaminobenzyl chitosan has been synthesized by Hakimi et al. [113]. Although the authors claimed that the modified chitosan had better water-solubility profile and potential for drug delivery, in their work, apart from cytotoxicity, they did not perform any studies related to the application of this type of thiolated chitosan as a mucoadhesive polymer. Clearly, this chitosan derivative will be of interest for evaluation of its mucoadhesive properties. 

### 4.4. Acrylated Chitosan

The use of acrylate groups in the development of mucoadhesive materials was pioneered by Davidovich-Pinhas and Bianco-Peled [114]. The mechanism of mucoadhesion is believed to be due to Michael-type addition reaction between the acrylate vinyl groups of the polymers and the sulfhydryl groups of mucus glycoproteins. The nature of this interaction was proved by ^1^H-NMR study, where the intensity of the peaks related to the vinyl groups of polyethylene glycol diacrylate hydrogels was decreased after their reactions with mucin dispersion [114]. Thus, the presence of covalent interactions with mucus is a common feature of acrylated and thiolated mucoadhesive materials [25,36,37,86,114,115,116,117]. The idea of acrylated chitosan synthesis was developed by Ma et al. [118]. However, they did not demonstrate any application in the mucosal drug delivery. This chitosan derivative is water-soluble, can be cross-linked under ultraviolet light using photoinitiator 2959 and has less antimicrobial activity compared to parent chitosan [118].

**Figure 11:**
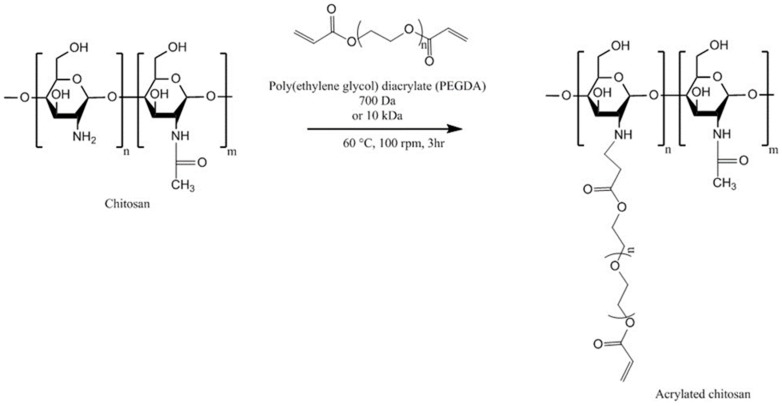
Synthetic pathway to acrylated chitosan [119].

Shitrit and Bianco-Peled [119] synthesized acrylated chitosan by reacting chitosan solution (1% *w*/*v* in 2% *v*/*v* acetic acid, molecular weight 207 kDa, degree of deacetylation 77.6%) with poly(ethylene glycol) diacrylate (PEGDA) via Michael-type reaction (Figure 11). Two different molecular weight PEGDA (0.7 and 10 kDa) were used. The acrylated chitosan was characterized using ^1^H-NMR spectroscopy and ninhydrin test. It was found that using smaller molecular weight (0.7 kDa PEGDA) at chitosan/PEGDA ratio of 1:4 resulted in more acrylation (98%) than using higher molecular weight PEGDA (10 kDa, 30%). The authors believed that this could be due the presence of greater molar amount of acrylate groups leading to a more efficient reaction. However, using chitosan/PEGDA 1:2 molar ratio led to the formation of a product with a lower degree of acrylation (45%).

The mucoadhesion was evaluated using tensile strength and rotating cylinder method using tablets of chitosan, thiolated and acrylated chitosan on porcine intestinal mucosa. The order of detachment force was the following: chitosan-PEGAc (10 kDa) ˃ thiolated chitosan ˃ chitosan = chitosan-PEGAc (0.7 kDa). Unexpectedly, the maximum detachment force of chitosan-PEGAc (0.7 kDa) was not significantly different from chitosan tablets. Both chitosan-PEGAc (10 kDa) and thiolated chitosan remained attached to the intestinal mucosa for more than 6 h, whereas chitosan-PEGAc (0.7 kDa) detached after 1 min. Chitosan tablets detached after 1.1 ± 0.2 h. The authors claimed that chitosan-PEGAc (0.7 kDa) has greater degree of acrylation than chitosan-PEGAc (10 kDa) and this means higher grafting density of PEG, which could result in the steric hindrance and preventing the covalent bonding with the cysteine-rich domain of mucus [119]. Similar trend with polyacrylic acid was observed; 450 kDa showed a stronger interaction with porcine gastric mucin whereas 2 kDa did not exert any effect [120]. Additionally, shorter PEG (smaller molecular weight) cannot deeply penetrate the mucosal tissues and results in a lower mucoadhesive strength, since mucoadhesive properties of polymers are proportional to the molecular weight [119]. Other studies reported that an optimum molecular weight of polymers is required to achieve maximal mucoadhesion. Small molecular weight polymers form weak gels and easily dissolve whereas high molecular weight polymers do not readily hydrate, thus the free binding groups are not available to interact with the mucus components. Therefore, in both cases, weak mucoadhesion can be observed [121].

### 4.5. Half-Acetylated Chitosan

Half-acetylated chitosan is another type of chitosan derivatives, which can be prepared by reacting chitosan with acetic anhydride. Several studies explored the solubility of half-acetylated chitosan and its subsequent effect on the antimicrobial and mucoadhesive properties of chitosan [15,17,35,122]. Qin et al. [122] found that half-acetylated chitosan had no antimicrobial activity against Staphylococcus aureus, Escherichia coli and Candida albicans. However, unmodified chitosan had antimicrobial effects against these microorganisms. They claimed that chitosan can interact with the components of the microorganism surfaces and thus be absorbed on their surfaces. Since the pH of bacterial and fungal cells is around 7, unmodified chitosan precipitates and forms an impermeable layer around the cells. This layer blocks the channels, which are essential for the cells survival. However, half-acetylated chitosan fully dissolved at neutral pH, thus did not form an impermeable layer, and led to a better survival of cells compared to unmodified chitosan.

Sogias et al. [17] demonstrated that half-acetylated chitosan (the degree of acetylation = 52 ± 4 mol %) was soluble over a broad pH range and did not precipitate below pH 7.4. This improved solubility profile of half-acetylated chitosan over unmodified chitosan was related to the reduced crystallinity (caused by disruption of inter- and intra-molecular hydrogen bonds) upon *N*-acetylation [15,17]. In another study, Sogias et al. [15] found that, at pH 2, half-acetylated chitosan interacted with porcine gastric mucin particles at a higher polymer/mucin ratio than unmodified chitosan, which was due to the decrease in the number of free amino groups in half-acetylated chitosan. At this pH, the amino groups undergone protonation and were responsible for the electrostatic interaction between chitosan macromolecules and mucin. They also revealed that at pH 7, where unmodified chitosan precipitates, half-acetylated chitosan was still able to interact with mucin particles. To explore the mechanisms of mucoadhesion, the polymer-mucin interaction was studied in the presence of sodium chloride (0.2 M), urea (8 M) and ethanol (10% *v*/*v*). These agents are known to disrupt the electrostatic interaction, hydrogen bonding and hydrophobic effects, respectively. The results indicated that all these forces were involved in the mucoadhesion of chitosan and half-acetylated chitosan. In case of half-acetylated chitosan, at pH 7, the electrostatic interaction was the major contributing force in the mucoadhesive interactions. This may be due to the higher negative charge density of mucin particles at pH 7 compared to pH 2 [15]. However, the mucoadhesive properties of unmodified chitosan at pH 7 were not evaluated, which could be due to its insolubility at this pH.

**Figure 12:**
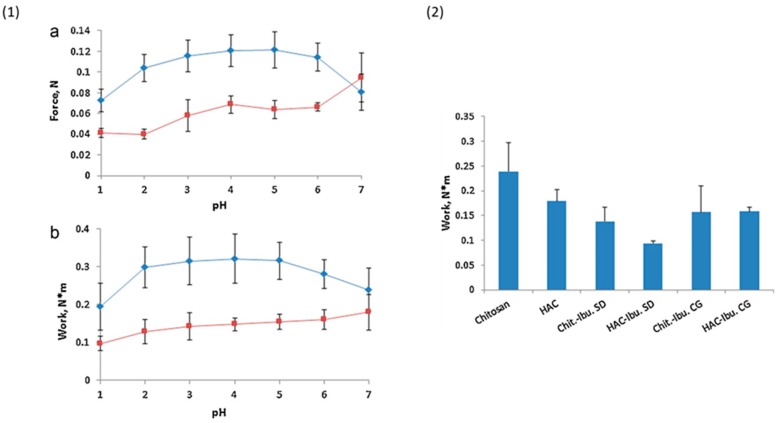
(**1**) Detachment force (**a**) and work of adhesion (**b**) for chitosan (♦) and half-acetylated chitosan (HACHI) (■) tablets as a function of pH on porcine gastric mucosal tissues at 37 ± 0.1 °C. Mean ± SD, *n* = 3. (**2**) Work of adhesion of tablets on porcine gastric mucosa at pH 7.0 and 37 ± 0.1 °C. Chit.: chitosan, Ibu.: ibuprofen, SD: spray-dried, CG: co-ground. Mean ± SD, *n* = 3. Reprinted from [35] with permission of Elsevier.

Sogias et al. [35] prepared microparticles containing ibuprofen and either chitosan or half-acetylated chitosan by two different techniques; spray-drying and co-grinding. 65 mg tablets were prepared from spray-dried chitosan and half-acetylated chitosan, spray-dried mixtures of chitosan or half-acetylated chitosan with ibuprofen and co-ground mixtures of the polymers and the drug. It was found that tablets of half-acetylated chitosan significantly enhanced ibuprofen release at pH 7. The force of detachment between unmodified chitosan tablets and porcine gastric mucosa was decreased when measured at very acidic (pH 1) and neutral (pH 7) media (Figure 12). However, the mucoadhesion of half-acetylated chitosan tablets was only decreased at low pH and increased linearly up to pH 7. Half-acetylated chitosan tablets were generally less mucoadhesive than chitosan tablets. This could be due to the reduction of cationic charge density upon acetylation, which diminished the electrostatic interaction with mucin [35]. Incorporation of ibuprofen in chitosans tablets resulted in a significant drop of mucoadhesion (Figure 12).

### 4.6. Glycol Chitosan

Glycol chitosan is a hydrophilic chitosan derivative, which can be prepared by adding ethylene glycol groups to chitosan backbone. It is soluble in water at any pHs [123,124]. It is commercially available from Sigma-Aldrich.

Glycol chitosan has been used in the design of nanoparticles for the delivery of poorly water- soluble drugs. Trapani et al. [124] prepared 6-coumarin-loaded glycol chitosan-TPP nanoparticles using ionic gelation method. Different cyclodextrins were used to form an inclusion complex with this dye. It was found that nanoparticles containing (2,6-di-*O*-methyl)-β-cyclodextrin could be internalized by Caco2 cells, which could be due to the mucoadhesive nature of chitosan. 

Glycol chitosan has been modified to prepare amphiphilic chitosan derivatives. Below, we will discuss two examples of these amphiphilic glycol chitosan derivatives.

#### 4.6.1. Palmitoyl Glycol Chitosan

Palmitoyl glycol chitosan is a hydrophobically-modified glycol chitosan. Its use in drug delivery started since the 1990s.The presence of both of hydrophilic and hydrophobic groups imparts it an amphiphilic character [125,126]. It has ability to self-assemble into vesicles suitable for delivery of water-soluble drugs such as bleomycin [126]. Its quaternized form (quaternary ammonium palmitoyl glycol chitosan) can self-assemble into micelles with a high drug loading capacity. It also facilitated transport of hydrophobic drugs including griseofulvin and propofol and hydrophilic drugs (but to a lower degree) including ranitidine through biological barriers such as intestinal and blood brain barriers, respectively, led to enhanced bioavailability [127,128]. It is conceivable that, the hydrophilic groups (–OH and –NH_2_) of glycol chitosan located in the external shell of the micelles and the hydrophobic groups in the cores. Thus, the mucoadhesive property of glycol chitosan should be well maintained upon self-assembly as these groups are mainly responsible for the mucoadhesive nature of chitosan and its derivatives [15,129].

**Figure 13:**
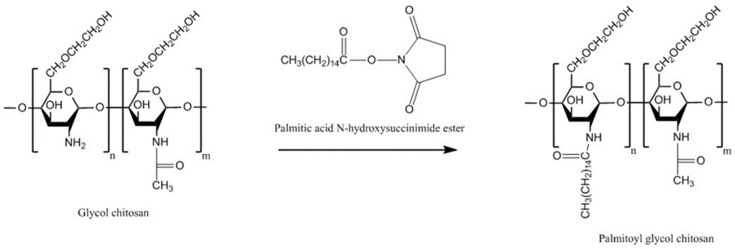
Synthetic pathway to palmitoyl glycol chitosan [130].

The hydrophobicity is one of the important factors affecting the mucoadhesive character of materials. Martin et al. [130] investigated this by synthesizing palmitoyl glycol chitosan with various degrees of palmitoylation (a hydrophobic group). First, glycol chitosan was dissolved in water before sodium bicarbonate and absolute ethanol were added. To this, ethanolic solution of palmitoyl-*N*-hydroxysuccinimide was added and then the mixture was stirred for 72 h in the dark (Figure 13). This was followed by dialysis and recovery of the product. The physically crosslinked gels were prepared by freeze drying the products and evaluated for their bioadhesive strength by measuring the force necessary to detach the gels from porcine buccal mucosa. It was found that by increasing the hydrophobicity (represented by the degree of palmitoylation), the hydration and erosion of the gels decreased. On the other hand, bioadhesion could be enhanced by increasing the hydrophobicity. Although no comparison with chitosan has been shown, palmitoyl glycol chitosans were found to be less bioadhesive than hydroxypropylmethyl cellulose/carbopol control. The most hydrophobic palmitoyl glycol chitosan gel (20.31 ± 2.22 mol % palmitoylation) resulted in the slowest controlled release of the model hydrophilic drug (FITC-dextran).

Siew et al. [127] developed nanoparticles based on quaternary ammonium palmitoyl glycol chitosan, which enhanced the oral absorption of both hydrophilic (ranitidine) and lipophilic drugs (griseofulvin and cyclosporine A). The bioavailability enhancement was believed to be due to a combination of increased drug dissolution rate (as a result of a great surface area of drug-loaded nanoparticles) and the mucoadhesive nature of chitosan, which increased the intestinal residence time of the nanoparticles, bringing them in close contact with the absorptive epithelial cells and thereby reducing the absorption barrier of the mucosal membrane [127]. This is because the established adhesion of the nanoparticles to the mucus layer provides some degree of penetration into the mucosal membranes [2].

#### 4.6.2. Hexanoyl Glycol Chitosan

**Figure 14:**
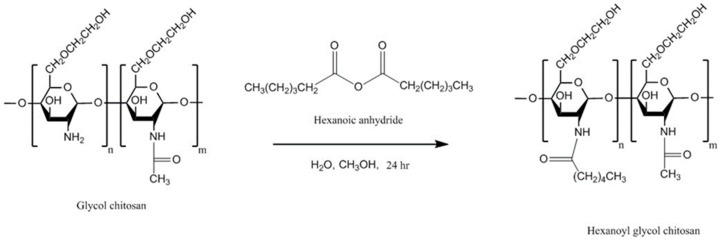
Synthetic pathway to hexanoyl glycol chitosan [131].

Cho et al. [131] synthesized hexanoyl glycol chitosan by *N*-acylation of glycol chitosan (Figure 14). To do that, glycol chitosan was dissolved in water and then diluted with methanol. Then, various amounts of hexanoic anhydride were added and the reaction mixture was continuously stirred for 24 h. The hexanoyl-glycol chitosan was precipitated by acetone and the product was recovered by lyophilization after been dialyzed against water. 

**Figure 15:**
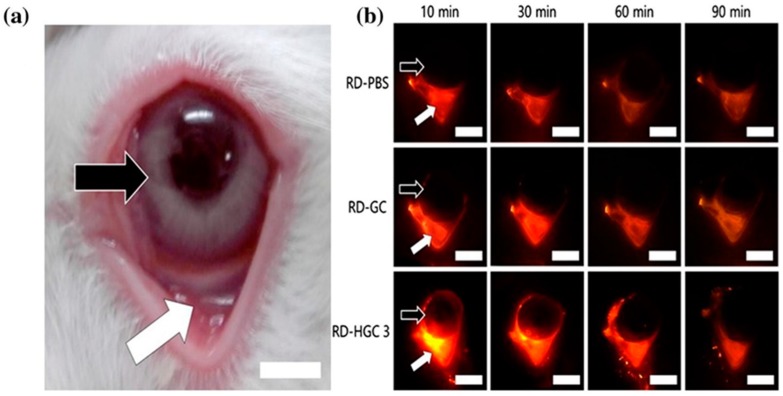
Photograph of rabbit eyes showing the eyeball and the inferior fornix (**a**). The fluorescence images of rabbit eyes at different time intervals after ocular administration of rhodamine-loaded PBS (RD-PBS), glycol chitosan (RD-GC) and hexanoyl glycol chitosan with 39.5 ± 0.4% degree of hexanoylation (RD-HGC 3). The eyeball and the inferior fornix (into which the formulations were administered) were shown by the black and white arrows, respectively (**b**). Scale bars = 5 mm. Reprinted from [131] with permission of Elsevier.

Interestingly, hexanoyl glycol chitosan with 39.5 ± 0.4% degree of hexanoylation had a thermosensitive gelling property as it underwent gelation at 37 °C. The in vitro release study showed no significant difference between brimonidine-loaded hexanoyl glycol chitosan-based formulation and the marketed eye drops (Alphagan P). However, the in vivo pre-ocular (inferior fornix of the eyes) retention study in rabbits revealed that hexanoyl glycol chitosan enhanced the retention of rhodamine in the pre-ocular tissues (Figure 15). The fluorescence signal from rhodamine was still strong after 60 min post administration, and became weak after 90 min. On the other hand, weak fluorescence signal was observed after only 10 min (and become weaker after 60 min) when both PBS (negative control) and unmodified glycol chitosan were used indicating their poor retention in pre-ocular tissues (Figure 15). Additionally, the intra-ocular pressure was significantly dropped and the therapeutic action was prolonged compared to unmodified glycol chitosan as well as conventional eye drops [131].

**Figure 16:**
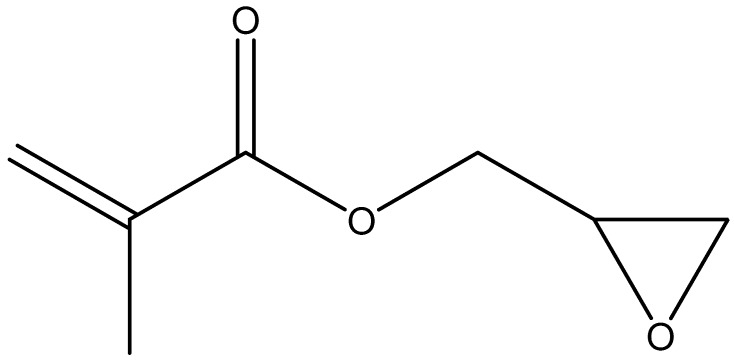
Chemical structure of glycidyl methacrylate.

Subsequently, Cho et al. [132] have further modified hexanoyl glycol chitosan by reacting it with glycidyl methacrylate (Figure 16) to form methacrylated hexanoyl glycol chitosan, which demonstrated a thermo-reversible sol–gel transition behavior in aqueous solutions. Moreover, the thermally-induced hydrogels could be chemically crosslinked by photo-crosslinking under UV-radiation. Although no studies, to our knowledge, reported the mucoadhesive potential of methacrylated hexanoyl glycol chitosan, the presence of a methacrylated part within this polymer can potentially lead to a strong interaction with the mucin because of the covalent bonding between methacrylate part of methacrylated hexanoyl glycol chitosan and the thiol groups of the mucin components.

### 4.7. Chitosan Conjugates

#### 4.7.1. Chitosan-Enzyme Inhibitors

These systems have been developed to protect orally administered peptide-based drugs from enzymatic degradation in the gastrointestinal lumen. Some mucoadhesive polymers including carbomer could also act as weak enzyme inhibitors [133], however, chitosan lacks this property. Examples of enzyme inhibitors include antipain, chymostatin, elastatinal and Bowman-Birk inhibitor [134]. It has been shown that enzyme inhibitors are toxic to certain types of cells. They also could induce pancreatic secretion of secretin and cholecystokinin in rats [135]. These characters could limit the application of free enzyme inhibitors in the formulation of peptide-based drugs. However, covalent attachment of enzyme inhibitors to mucoadhesive polymers such as chitosan could reduce the unwanted effects as their absorption can be reduced. Bernkop-Schnürch et al. [13] synthesized chitosan-antipain conjugate. The synthetic approach based on the formation of amide bond between carboxylic acid groups of enzyme inhibitors and the primary amino groups of chitosan which was mediated with EDAC and sulfo-*N*-hydroxysuccinimide. Chitosan-antipain conjugate not only showed mucoadhesive properties similar to unmodified chitosan, it also inhibited the action of trypsin. Tablets containing 5% chitosan conjugate protected insulin from trypsin inactivating effect. A sustained insulin release for 6 h was also achieved.

#### 4.7.2. Chitosan-Complexing Agent

Ethylenediaminetetraacetic acid (EDTA) is a potent chelating agent and has US FDA approval for the treatment of heavy metal poisoning since 1950s [136]. Removal of ions has been shown to enhance the permeation of antiviral drugs such as dolutegravir across Caco2 cells monolayer and rat intestinal mucosa ex vivo [137]. EDTA is also able to decrease pre-systemic metabolism of peptide-based drugs by inhibiting brush border membrane bound enzymes by their deprivation of ions such as Zn^2+^ in the mucous membrane [134,138]. However, the rapid biodistribution of EDTA limits this application. Thus, chitosan-based EDTA system has been developed which has mucoadhesive properties on one side and metal chelating ability on the other side [138,139]. 

Compared to unmodified chitosan, chitosan-EDTA tablets showed better retention on porcine intestinal mucosa. The mucoadhesive strength decreased with the reduction of the % of EDTA attached to chitosan. It also inhibited Zn- and Co-dependent proteases including carboxypeptidase A and aminopeptidase N. This is because chitosan-EDTA conjugate strongly bound to Zn and Co. [140].

**Figure 17:**
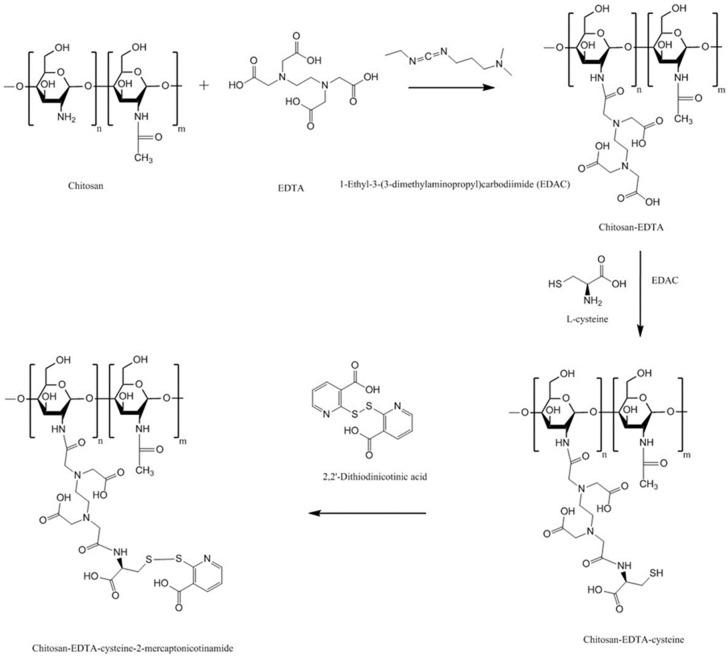
Synthetic pathway to chitosan-EDTA-cysteine-2-mercaptonicotinamide [138].

**Figure 18:**
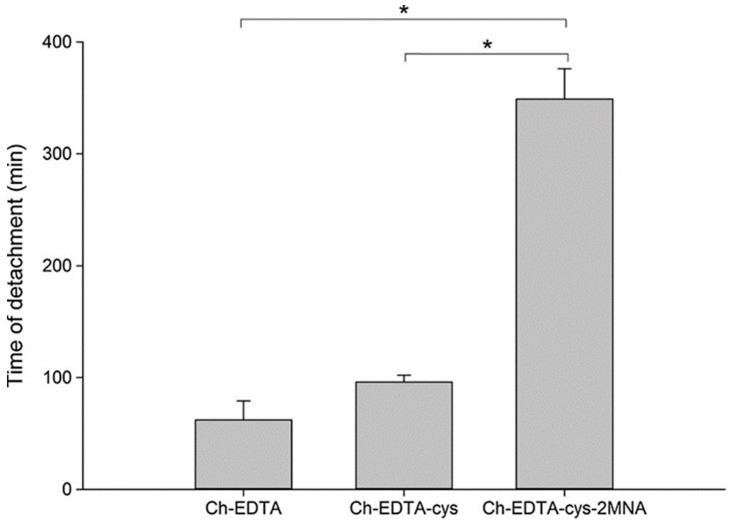
Mucoadhesion time of mini-tablets containing 30 mg of Ch-EDTA, Ch-EDTA-cys or Ch-EDTA-cys-2MNA studied by rotating cylinder method using porcine intestinal mucosa. Ch: chitosan, cys: cysteine, 2MNA: 2-mercaptonicotinamide. (Mean ± SD, *n* = 5, * denotes statistical significant difference at *p* < 0.05). Reprinted from [138] with permission of Elsevier.

S-protected thiolated chitosan-EDTA has also been synthesized to combine the advantages of EDTA, thiolation and pre-activation or protection of thiol groups. The synthetic pathway is shown in Figure 17 [138]. The multifunctional thiolated chitosan exhibited 5.6- and 3.6-fold longer residence time on porcine intestinal mucosa compared to chitosan-EDTA and chitosan-EDTA-cysteine, respectively (Figure 18).

#### 4.7.3. Chitosan-EDTA-Enzyme Inhibitors

By combining enzyme inhibitors and complexing agents coupled with chitosan, the degradation of peptide-based drugs by the gut luminal enzymes could be significantly minimized [139]. Additionally, as EDTA could bind to ions such as Zn^2+^ and Ca^2+^, the concentration of free forms of these ions can be reduced. This decreases the formation of non-absorbable complexes between some drugs and these ions leading to enhanced drug permeation [136,137]. Thus, chitosan-EDTA-serine protease inhibitors were synthesized using a two-step approach. First, to form chitosan-serine protease inhibitors, covalent attachment of antipain, chymostatin and elastatinal to chitosan was performed. Second, chitosan-enzyme inhibitors were bound to EDTA. Tensile study using porcine intestinal mucosa demonstrated that the mucoadhesive strength of the chitosan-EDTA-serine inhibitor was lower than both chitosan-EDTA and chitosan. The reduction of the mucoadhesion of chitosan-EDTA-serine protease inhibitors could be due to the substitution of the free amino groups of chitosan or chitosan-EDTA upon covalent attachment to the enzyme inhibitors [139].

### 4.8. Chitosan-Catechol (Chi-C)

**Figure 19:**
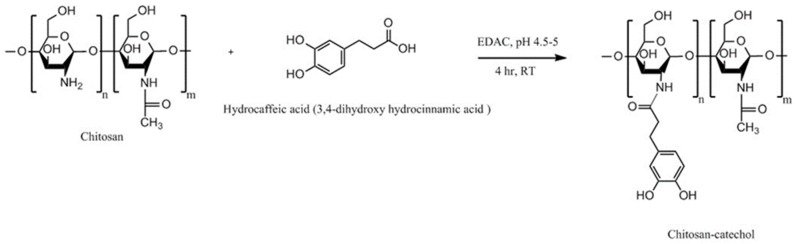
Synthetic pathway to chitosan-catechol using carbodiimide chemistry [141].

Catechol is a naturally occurring compound. It is an essential component of l-3,4-dihydroxyphenylalanine (l-DOPA), which is an amino acid secreted by certain marine mussels (e.g., Mytilus edulis), which have ability to adhere to various substrates under wet conditions [141]. This adhesive property is mainly linked to the ability of catechol to form covalent and non-covalent bonds to different organic, inorganic, and metallic surfaces [142,143]. Generally, chitosan-catechol can be synthesized by chemical, electrochemical and enzymatic methods. The chemical method includes three main approaches: amide bond formation using carbodiimide chemistry (Figure 19), reductive amination using aldehyde-terminated catechol and reducing agents such as NaCNBH_3_ or NaBH_4_, and formation of catechol-amine adducts using oxidizing agents such as NaIO_4_ [141,144].

**Figure 20:**
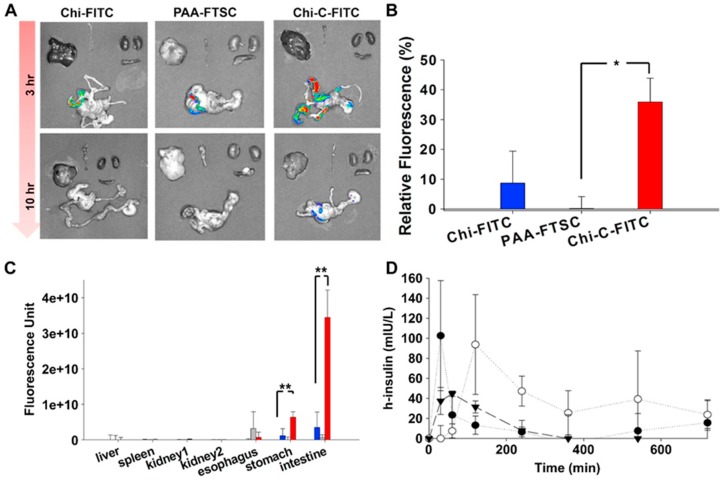
Chitosan-fluorescein isothiocyanate (Chi-FITC), polyacrylic acid-fluorescein-5-thiosemicarbazide (PAA-FTSC) and chitosan-catechol-fluorescein isothiocyanate (Chi-C-FITC) were orally administered to BALB/c mice and the animals were euthanized after 3 or 10 h. (**A**) The extracted organs were imaged using in vivo imaging system. (**B**) The relative fluorescence intensity of Chi-FITC, PAA-FTSC and Chi-C-FITC in the gastrointestinal tract (esophagus, stomach and intestine) at 10 h after administration. (**C**) The fluorescence in the liver, spleen, kidneys, esophagus, stomach, and small/large intestine at 10 h after administration are shown (mean ± SD, *n* = 3 mice/time point). (* denotes statistical significant difference at *p* < 0.05, ** indicates *p* < 0.005). (**D**) The human (h)-insulin (closed triangle), h-insulin/chitosan (closed circle) and h-insulin/chitosan-catechol (open circle) were orally administered to Wistar rats and blood insulin concentration was measured using enzyme-linked immunosorbent assay (ELISA) (*n* = 4 rats/time point). Reprinted from [141] with permission of Elsevier.

Inspired by mussel adhesion to surfaces, Kim et al. [141] synthesized chitosan-catechol conjugate by reacting chitosan with 3,4-dihydroxy hydrocinnamic acid mediated with EDAC (Figure 19). Mucoadhesion was evaluated in vitro using mucin-particle interaction, turbidimetry, surface plasmon resonance (SPR) spectroscopy and rheological characterization as well as in vivo fluorescence imaging technique and fluorescence measurement in various organs of mice. Chitosan-catechol conjugate showed superior mucoadhesion than both unmodified chitosan and polyacrylic acid. The in vivo study explored the difference in the retention of different polymers in different body sites. No fluorescence was detected in organs lacking mucosal tissues including liver, spleen, and kidney (Figure 20). However, at 3 h post-oral administration, strong fluorescence signal from chitosan-catechol conjugate in intestinal tissues was observed (Figure 20). This could be due to the formation of strong covalent bonds via Michael-type addition reaction upon the reaction of oxidized form of catechol (quinone) and amine or thiol functionalities of mucins or Schiff base formation reaction [141]. The electrostatic attractive interaction between the positively charged groups of chitosan and negatively charged carboxyl and sulfate groups of mucin could lead to an initial contact stage and the adsorption of chitosan-catechol macromolecules on the mucosal surfaces. This was then followed by an established consolidation stage via the covalent interaction [121,141]. Unmodified chitosan and polyacrylic acid showed poor fluorescence signal. The retention of chitosan-catechol conjugate decreased significantly in both stomach and esophagus. The authors claimed that chitosan-catechol conjugate-mucin interaction was stronger when the pH of mucin solution was 7 compared to pH 2 [141]. This might explain better retention in small intestine, where pH is near neutral compared to poor retention in stomach (highly acidic) and esophagus (slightly acidic, pH 4–6) [141]. The oxidation of catechol to quinone in alkaline environment is more likely than in acidic environment, which could provide additional adhesive interactions [141,142,145]. On the other hand, polyacrylic acid showed slightly greater mucoadhesion to esophagus than stomach and intestine (Figure 20C). The difference in the pH of these organs might explain this observation as it may affect the structures of both polyacrylic acid and the mucus layer resulting in a different nature and extent of mucoadhesive interactions at different pHs [146]. Some studies reported that the mucoadhesive nature of polyacrylic acid may be due to its ability to form hydrogen bonds with the mucus components [120,141,146,147], which is strongest at slightly acidic pHs, depending on the type of the polymer [147,148]. However, Kim et al. [141] suggested further studies to investigate the organ-specific mucoadhesive properties of chitosan, chitosan-catechol and polyacrylic acid. Chitosan-catechol conjugate also enhanced the oral bioavailability of insulin and C_max_ reached after 2 h compared to unmodified chitosan which was 30 min (Figure 20D).

### 4.9. Methyl Pyrrolidinone Chitosan

**Figure 21:**
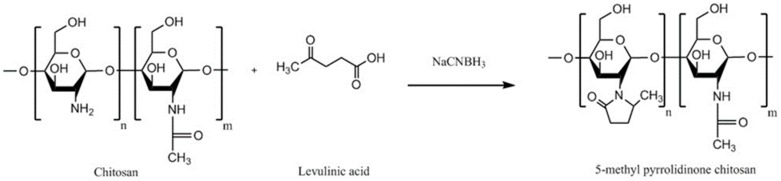
Synthetic pathway to 5-methyl pyrrolidinone chitosan.

Methyl pyrrolidinone chitosan can be synthesized by reacting chitosan with levulinic acid (Figure 21) [149,150]. Specific experimental conditions including pH of the reaction mixture, type and the rate of addition of reducing agents (NaCNBH_3_ or NaBH_4_), molar ratio of levulinic acid/chitosan/reducing agents are required to obtain methyl pyrrolidinone chitosan and not *N*-carboxybutylchitosan derivatives [151,152]. Sandri et al. [153] studied the mucoadhesive and penetration enhancing properties of various chitosans including 5-methyl pyrrolidinone chitosan, low molecular weight chitosan, a partially re-acetylated chitosan and chitosan HCl using buccal or submaxillary bovine mucin dispersion, vaginal mucosa or porcine gastric mucin dispersion. It was found that different chitosans behaved differently in different substrates. In submaxillary mucin dispersion, chitosan·HCl was the most mucoadhesive. However, 5-methyl pyrrolidinone chitosan showed the greatest mucoadhesion among other polymers in all other studied substrates and provided the greatest permeation of acyclovir through porcine cheek mucosa and deepest penetration into the vaginal mucosa. This could be due to the penetration enhancing effect of 5-methyl pyrrolidinone, which has been demonstrated in other studies [154,155].

### 4.10. Cyclodextrin-Chitosan

Cyclodextrins can enhance solubility and dissolution of poorly water-soluble drugs by forming inclusion complexes. In 2001, the idea of grafting cyclodextrin to chitosan was adopted by Auzély-Velty and Rinaudo [156], who used a reductive amination approach, where a solution of chitosan in acetic acid/methanol was reacted with aldehyde-containing cyclodextrin derivative in the presence of sodium cyanoborohydride (NaCNBH_3_). The reaction was mediated with EDAC. The inclusion ability of the grafted-cyclodextrin was studied using NMR spectroscopy and found that it could form inclusion complexes with two model compounds tert-butylbenzoic acid and (+)-catechin.

**Figure 22:**
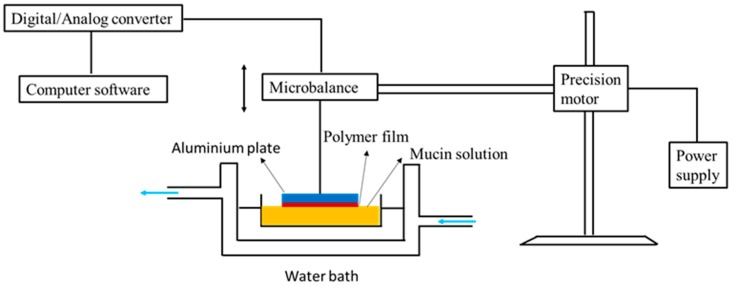
Experimental set-up for evaluation of mucoadhesion using microbalance method according to Venter et al. [157] with some modifications.

In 2006, Venter et al. [157] studied the mucoadhesion of this cyclodextrin-chitosan derivative by tensile separation test (microbalance method) using partially purified porcine gastric mucin type III (Sigma, UK) as a substrate. Figure 22 shows the experimental set-up for the mucoadhesion study. Briefly, the aluminum plates of the apparatus were coated with the polymer solution (1% *w*/*v*) and left to dry until polymeric films formed. Mucin solution (30% *w*/*v*) was prepared and placed in a water bath (25 °C). The polymer-coated plate was lowered to contact with the mucin solution for 2 min. Then, the maximum detachment force to separate the polymeric films from the mucin solution was measured using a computerized system. It was found that upon derivatization, chitosan lost its mucoadhesive properties by 13.5%, but, it was 12% stronger than pectin.

In another study, Chaleawlert-umpon et al. [158] synthesized citrated cyclodextrin-*g*-chitosan. In this study, citric acid was used to facilitate cyclodextrin mobility. Glycidyl trimethylammonium chloride was also used to quaternize chitosan. The mucoadhesion study using mucin-particle interaction method and SPR revealed that combination of quaternization and citrate modification led to a significant enhancement in the mucoadhesive interactions. This could be due to an increase in the cationic charge of chitosan as well as hydrogen bonding between carboxyl and hydroxyl groups of the spacer and the mucus components.

### 4.11. Oleoyl-Quaternised Chitosan

Yostawonkul et al. [159] developed a nanostructure lipid carrier for the delivery of lipophilic drug molecules using high-pressure homogenization technique. They found coating of these carriers with oleoyl-quaternised chitosan enhanced carcinoma Caco-2 cellular uptake of the model drug (alpha-mangostin). This enhancement could be due to the mucoadhesive properties of oleoyl-quaternised chitosan, which was evaluated by mucin-particle interaction method. However, cytotoxicity of the carriers was also increased and thus the authors suggested careful optimization of the drug loading to target cancer cells for chemotherapy.

## 5. Comparison of Different Chitosan Derivatives

Table 1 illustrates the advantages and disadvantages of different chitosan-based systems reported in the literature together with the drug model, administration routes and mucus substrates types that were used to evaluate them.

## 6. Conclusions

In this review, general methods of synthesis of potential mucoadhesive chitosan derivatives have been highlighted. Some properties of chitosan and chitosan derivatives have been discussed. These include solubility profile, stability, mucoadhesive and permeation enhancing effects. The mucoadhesive properties of the derivatives have been particularly considered. It was shown that the mucoadhesive properties of some derivatives have been significantly increased compared to unmodified chitosan. In the majority of cases, this resulted in an enhancement in the bioavailability and a significant improvement of the therapeutic efficacy of several candidate drugs compared to unmodified chitosan. In some others, the mucoadhesive character either did not change or slightly decreased. This however, was compensated with an improvement of other important chitosan properties including solubility in physiological pH and cohesiveness, which are crucial parameters in mucoadhesion. Therefore, improvement in the properties of chitosan derivatives discussed in this review clearly demonstrate that its chemical modification could potentially lead to further advances in transmucosal drug delivery. However, chemical modification of chitosan has limitations. These include low reproducibility, especially with hydrophobically-modified chitosans, poor solubility of chitosan in organic solvents used for the synthesis and changes with the degree of acetylation during chemical modification.

## Figures and Tables

**Table 1:** A summary of chitosan derivatives properties with examples of drug candidates used in the mucoadhesive drug delivery evaluation.

Chitosan Derivatives	Advantages	Disadvantages	Drug	Route of Administration/Substrate	References
Trimethyl chitosan	Soluble at broad range of pHs (2–12), strong mucoadhesion; decreased TEER; increased paracellular permeability of basic or neutral macromolecules	Strong aggregation with anionic macromolecules such as heparin	Buserelin, ropinirole·HCl	Oral, small intestine, cattle nasal mucosa	[52,160,161]
*N*-carboxymethyl chitosan	Decreased TEER; increased paracellular permeability of anionic macromolecules	Insoluble at pH 3–7 (depending on the degree of substitution) due to its polyampholytic character	Low molecular weight heparin; Ofloxacin	Oral, rat small intestine; Ocular, rabbit eyes, in vivo	[73,76,82]
Chitosan-cysteine	Same mucoadhesion as unmodified chitosan, improved cohesion compared to unmodified chitosan, permeation enhancing effect	Susceptible to premature oxidation, undesirable side reactions led to the formation of (chitosan-cysteine-cysteine)_n_ side chains	-	Oral, porcine intestinal mucosa	[25,84]
Chitosan-*N*-acetylcysteine	50-fold longer retention time than unmodified chitosan, biodegradability as indicated by the reduction of its solution viscosity after addition of hen white egg	Susceptible to premature oxidation	-	Oral, flat faced-discs, porcine intestinal mucosa	[87]
Chitosan-TGA	Controlled drug release, longer disintegration time (up to 100-fold) and 26-fold longer mucoadhesion time against unmodified chitosan	Need of mediator such as EDAC	Clotrimazole	Vaginal, tablets, bovine vaginal mucosa	[162]
Chitosan-TBA	Strong mucoadhesion, permeation enhancing effect, controlled release, no need for mediator	Prone to oxidation. In addition, unintended cyclisation side reactions	Insulin, cefadroxil	Oral, tablets, porcine and rat intestinal mucosa	[108,163]
Chitosan-thioethylamidine	Much quicker synthetic reaction rate than chitosan-TBA (1.5 h vs. 24 h), 8.9-fold longer mucosal detachment time than unmodified chitosan, controlled release, no cyclisation side reactions as in chitosan-TBA	Stability issues	FITC-dextran	Oral, tablets, porcine intestinal mucosa	[88]
Chitosan-glutathione	Improved stability compared to unmodified chitosan, enhanced mucoadhesion (9.9-fold increased adhesion force and 55-fold longer adhesion time), 4.9-fold higher permeation-enhancing effect against unmodified chitosan, used as oxidative stress suppressant	Stability issues	Thymopentin	Oral, tablets, in vitro porcine rat intestinal mucosa; Oral nanoparticles, in vivo rats; Injectable hydrogels	[89,91,104]
Pre-activated (S-protected) thiolated chitosan	Improved stability and mucoadhesion compared to unmodified chitosan and unprotected thiolated chitosan	2-fold less swelling than unmodified chitosan	Leuprolide; Antide	Oral, tablets, porcine intestinal mucosa Oral, rat intestinal mucosa	[111,112]
Acrylated chitosan	Strong mucoadhesion, water-soluble	Use of low molecular weight PEGDA results in a weaker mucoadhesion	-	Oral, porcine intestinal mucosa	[119]
Half-acetylated chitosan	Better solubility at higher pHs (up to 7.4) compared to unmodified chitosan, sustained drug release	Less mucoadhesive compared to unmodified chitosan	Ibuprofen	Oral, porcine gastric mucosa	[35]
Palmitoyl glycol chitosan	Amphiphilic property, diminished erosion and slow hydration led to controlled release, control bioadhesive strength by changing the degree of palmitoylation	Potential problems with reproducibility with the degrees of substitution related to insolubility of the final product	FITC-dextran	Buccal/disc shaped gels, porcine buccal mucosa	[130]
Hexanoyl glycol chitosan	In situ gelling property, in vivo ocular retention, longer duration of action	-	Rhodamine, brimonidine	Ocular, rabbit, in vivo ocular tissues	[131]
Chitosan-enzyme inhibitors	Protects drugs from enzymatic degradation. Controlled antipain release over 6 h, mucoadhesive properties preserved	Potential stability issues	Insulin	Oral, flat-faced discs, porcine intestinal mucosa	[13]
Chitosan-EDTA	Better mucoadhesion than unmodified chitosan Inhibits Zn and Co-dependent proteases including carboxypeptidase A and aminopeptidase N	No Ca-dependent serine proteases inhibition	-	Oral, flat-faced discs, porcine intestinal mucosa	[140]
Chitosan-enzyme inhibitors-EDTA	Strong inhibitory action against serine proteases, Zn-dependent exopeptidases including carboxypeptidase A and B, aminopeptidase N	Less mucoadhesive than unmodified chitosan and chitosan-EDTA	-	Oral, flat-faced discs, porcine intestinal mucosa	[139]
Chitosan-catechol conjugate	Strong mucoadhesion, higher solubility at neutral pH, sustained drug release, improved therapeutic effect in vivo compared to unmodified chitosan	Poor mucoadhesion in acidic environment, optimum degree of substitution (7.2%) is required to achieve water-soluble product and formation of large gel-like aggregates has been observed for greater degree of substitution (12.7%)	Lidocaine; Sulfasalazine	Oral, mice gastrointestinal tract, porcine gastric mucin type II; Buccal, hydrogels, porcine and rabbit buccal mucosa; Rectal, hydrogels, mice rectal mucosa in vivo	[141,143,164,165]
Methyl pyrrolidinone chitosan	Greater mucoadhesion and penetration enhancing effect than unmodified chitosan	-	Acyclovir	Buccal and vaginal, porcine cheek or submaxillary bovine mucin, vaginal mucosa, or porcine gastric mucin	[153]
Chitosan-cyclodextrin	Inclusion ability, sustained release	Weaker mucoadhesion than the parent chitosan	-	Porcine gastric mucin	[156,157]
